# Association of Glomerular Filtration Rate and Carotid Intima-Media Thickness in Non-Diabetic Chronic Kidney Disease Patients over a 4-Year Follow-Up

**DOI:** 10.3390/life11030204

**Published:** 2021-03-05

**Authors:** Azer Rizikalo, Slavica Coric, Andrija Matetic, Mirjana Vasilj, Zoran Tocilj, Josko Bozic

**Affiliations:** 1 Department of Urology, University Clinical Hospital Mostar, 88000 Mostar, Bosnia and Herzegovina; azer.r@skbm.ba; 2 Department of Nephrology, University Clinical Hospital Mostar, 88000 Mostar, Bosnia and Herzegovina; slavica.c@skbm.ba; 3 Department of Cardiology, University Hospital of Split, 21000 Split, Croatia; amatetic@kbsplit.hr; 4 Department of Cardiology, University Clinical Hospital Mostar, 88000 Mostar, Bosnia and Herzegovina; mirjana.v@skbm.ba; 5 Sports Medical Centre “Diomed”, 21000 Split, Croatia; zorantocilj@diomed.hr; 6 Department of Pathophysiology, University of Split School of Medicine, 21000 Split, Croatia

**Keywords:** carotid intima-media thickness, glomerular filtration rate, chronic kidney disease

## Abstract

Patients with chronic kidney disease (CKD) have increased risk of cardiovascular events. However, the association of glomerular filtration rate (GFR) and carotid intima-media thickness (CIMT) in non-diabetic CKD patients is under-investigated. This prospective study was conducted at University Clinical Hospital Mostar over a 4-year period and enrolled a total of 100 patients with stage 2 and 4 CKD (50 patients per group). Stage 4 CKD group had significantly higher baseline CIMT values (1.13 ± 0.25 vs. 0.74 ± 0.03 mm, *P* < 0.001), and more atherosclerotic plaques at the study onset (13 (26%) vs. 0 (0%), *P* < 0.001) compared to stage 2 CKD. A statistically significant 4-year increase in GFR (coefficient of 2.51, 3.25, 2.71 and 1.50 for 1-year, 2-year, 3-year and 4-year follow-up, respectively, *P* < 0.05) with non-significant CIMT alterations has been observed in stage 2 CKD. Furthermore, linear mixed effects analysis revealed significant decrease in GFR (coefficient of −6.69, −5.12, −3.18 and −1.77 for 1-year, 2-year, 3-year and 4-year follow-up, respectively, *P* < 0.001) with increase in CIMT (coefficient of 0.20, 0.14, 0.07 and 0.03 for 1-year, 2-year, 3-year and 4-year follow-up, respectively, *P* < 0.001) in stage 4 CKD. GFR and CIMT showed significant negative correlation in both CKD groups during all follow-up phases (*P* < 0.001). Furthermore, multiple linear regression analysis revealed significant independent prediction of CIMT by baseline GFR (B = −0.85, *P* < 0.001), while there was no significant prediction of CIMT with other covariates. In conclusion, this study demonstrates significant association of GFR and CIMT in non-diabetic stage 2 and stage 4 CKD during the 4-year follow-up.

## 1. Introduction

Chronic kidney disease (CKD) is a progressive multifactorial disease characterized by permanent impairment of the renal function and/or structure [1]. It represents an emerging health burden with substantial morbidity and mortality [2]. Renal function in CKD can show diverse trends so that some patients develop a rapid decline in glomerular filtration rate (GFR) while others have stable function for years [3,4]. It has been shown that numerous factors have a role in this complex pathophysiologic cascade. Patients with CKD have an increased risk of cardiovascular events including atherosclerosis-related complications [5]. CKD initiates numerous pathophysiological processes that could represent a perpetual interaction of renal and cardiovascular diseases [6]. In fact, entities such as cardiorenal syndrome highlight the aforementioned close bidirectional relationship of renal, cardiac and vascular homeostasis [7]. In addition to the negative effects of CKD on cardiovascular homeostasis, a number of comorbidities such as type 2 diabetes represent risk factors for the development of cardiovascular and renal disorders [8]. One of the most commonly used techniques to assess atherosclerosis and overall cardiovascular risk in CKD patients is carotid ultrasound [9].

Carotid intima-media thickness (CIMT), an ultrasound measure, has been shown to predict future cardiovascular events in the general population [10], while emerging studies have tested its use in the CKD population [5,11,12,13,14]. Previous studies have mostly encompassed severe CKD [5,12,14], dialysis patients [11,15], renal transplant recipients [16], or pediatric CKD population [17,18,19,20]. Only a limited number of studies have assessed the association between renal function and atherosclerosis in patients with mildly impaired renal function [13,21,22]. However, the presence of different comorbidities such as diabetes mellitus, which is per se an independent predictor of atherosclerosis, represents a confounding issue [23,24]. There are scarce data regarding the association of GFR and CIMT in adult non-diabetic CKD patients. Therefore, the aim of this study was to assess the association of renal function and atherosclerosis, measured by eGFR and CIMT, in non-diabetic patients with stage 2 and 4 of CKD over a 4-year follow-up period. Finally, the goal was to determine the differences in the rates of CKD progression, hemodialysis initiation, newly developed atherosclerotic plaques and mortality, as well as the composite of the aforementioned events between the study groups.

## 2. Materials and Methods

### 2.1. Ethical Considerations

The study protocol was approved by the Ethics Committee of the University Clinical Hospital Mostar (Protocol number: 5115/13; Mostar, 26 September 2013). All procedures were performed in accordance with the Helsinki declaration and its amendments. Written informed consent was obtained from all individual participants included in the study.

### 2.2. Study Design and Protocol

**Figure 1:**
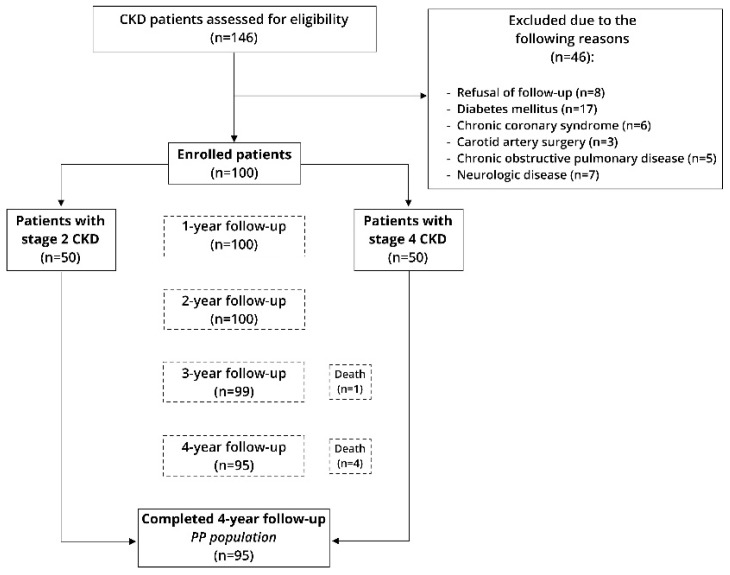
Flow diagram of the study. Legend: CKD–chronic kidney disease.

This prospective longitudinal study was conducted at the Department of Nephrology of the University Clinical Hospital Mostar over a period from December 2014 to December 2018. All participants were informed about the procedures, course, aim of the study and follow-up protocol. Each subject was assessed upon study onset and further at each study timepoint (every year) for a total of four years. Baseline measurements involved detailed clinical, physical and laboratory evaluation, while the follow-up evaluation involved only estimate glomerular filtration rate (eGFR) and CIMT measurements (Figure 1).

### 2.3. Subjects

A total of 146 consecutive adult patients with stage 2 and 4 of CKD undergoing medical evaluation at the outpatient clinic of the Department of Nephrology were assessed for eligibility, while 100 patients were finally included in the research (50 patients per group). The diagnosis of CKD and the study groups were defined according to international guidelines, and classified into five stages with regards to the residual GFR: stage 1 (90–120 mL/min/1.73 m²), stage 2 (60–89 mL/min/1.73 m²), stage 3a (45–59 mL/min/1.73 m²), stage 3b (30–44 mL/min/1.73 m²), stage 4 (15–29 mL/min/1.73 m²) and stage 5 (<15 mL/min/1.73 m²). Estimated GFR (eGFR) was calculated using the Modification of Diet in Renal Disease (MDRD) equation [25]. Exclusion criteria were diabetes mellitus; any form of dialysis prior to the study onset; severe cardiovascular, pulmonary, neurologic, or psychiatric disease; active malignant disease; active infection; pregnancy; life expectancy of less than 12 months; previously established cardiovascular event; carotid artery surgery; organ transplantation; and alcohol or substance abuse. All patients received angiotensin convertase inhibitors (ACE-I) or angiotensin II receptor blockers (ARB). Other medications have been used according to the clinical indication. Erythropoietin analogues were utilized for the management of anemia, and sevelamer phosphorus binders were used to treat hyperphosphatemia. All medications were titrated according to recommended dosing in different CKD groups.

### 2.4. Clinical Assessment and Anthropometric Measurements

Detailed medical history was obtained from the anamnestic data and electronic health records, and all subjects underwent a detailed physical examination including anthropometric data assessment. Anthropometric measurements were conducted according to the conventional medical standards. A calibrated medical scale with an altitude meter (SECA 700, Seca GmbH & Co. Hamburg, Germany) was used to measure body mass and height. BMI was calculated by dividing the value of body mass (kg) by the squared value of height (m^2^). Blood pressure was measured using a standard mercury sphygmomanometer, with a suitable arm, after the subject rested in a supine position for 15 min. Three measurements were performed with an interval of 10 minutes and the average measurements were taken as the final value of blood pressure.

### 2.5. Laboratory Analysis

Blood samples were collected from all participants included in the study, in the morning, after a 12-h fasting period. Laboratory analysis has been conducted following the standard laboratory methods. All samples were analyzed in a single laboratory by an experienced biochemist following the same standard procedure. Complete blood count (CBC) was determined by flow cytometry. Serum calcium and phosphorus were measured by a photometric staining test, while 25-hydroxyvitamin D (25(OH)D3) and parathyroid hormone (PTH) were determined with a microparticles chemiluminescence method (CMIA). Plasma C-reactive protein (CRP) levels were determined using a standard immunoturbidimetric method and represent standard CRP values. High density lipoprotein (HDL), low density lipoprotein (LDL) and triglycerides were determined using an enzyme staining test. Other biochemical analyses were measured by standard laboratory methods. Calcium levels were corrected according to albumin concentration, by increasing or decreasing total calcium levels for 0.8 units (mg/dL) with a decrease or increase of albumin levels for 1 unit (g/dL), respectively. The following formula was used: total calcium (mg/dL) + 0.80 × change in albumin from normal (g/dL) = corrected calcium (mg/dL).

### 2.6. Measurements of Carotid Intima-Media Thickness

The degree of atherosclerosis was assessed using an ultrasound of the common carotid arteries. A type B linear probe with a frequency range of 5–10 MHz was utilized (Philips iU 22, Philips, The Netherlands). Subjects were in a supine position and the carotid arteries were examined bilaterally in an oblique position from the front and rear. Three measurements were taken (0.5, 1 and 2 cm below the carotid bifurcation, respectively) by the same physician experienced in ultrasound imaging. The final CIMT value was calculated using an arithmetic mean of the bilateral measurements. Carotid plaque was defined as CIMT > 1.5 mm within the lumen of an artery which is in accordance with the European Mannheim consensus definition [26].

### 2.7. Statistical Analysis

Statistical software IBM SPSS Statistics (IBM Corp, Armonk, NY, USA; version 25) for Windows was used for statistical data analysis. Data were expressed as mean ± standard deviation or median (interquartile range) for continuous variables, and as whole numbers and percentage for categorical variables. The Kolmogorov–Smirnov test was used for normality of data distribution. Student’s t-test was used for the analysis of parametric continuous data, and Mann-Whitney U test was used for the analysis of non-parametric continuous data. Chi-square and Fisher’s test were used for analysis of categoric variables. Pearson’s correlation served for assessment of correlation between selected variables. Multivariable linear regression analysis with enter selection algorithm was used to determine the relative importance of independent variables (age, BMI, systolic blood pressure, LDL-cholesterol and CRP) in prediction of CIMT levels. The linear mixed effect model with the unstructured covariance structure and restricted maximum likelihood algorithm were used for the within-subjects comparison of the eGFR and CIMT over the follow-up. Results of the linear mixed effects model analysis were expressed as parameter estimates with 95% confidence interval (CI), t-value, Bayesian information criteria and the corresponding *P* value. The statistical significance was defined as *P* < 0.05.

## 3. Results

### 3.1. Subjects’ Characteristics

There was no significant difference in demographic and anthropometric characteristics between studied groups, except in age, systolic blood pressure and BMI which were higher in stage 4 CKD group (60 (53, 63) vs. 48 (36, 54) years, *P* < 0.001; 149.30 ± 18.24 vs. 127.80 ± 9.80 mmHg, *P* < 0.001; and 27.46 ± 3.36 vs. 22.56 ± 2.67 kg/m^2^, *P* < 0.001, respectively). Other baseline characteristics are shown in Table 1.

### 3.2. eGFR and CIMT Values

**Figure 2:**
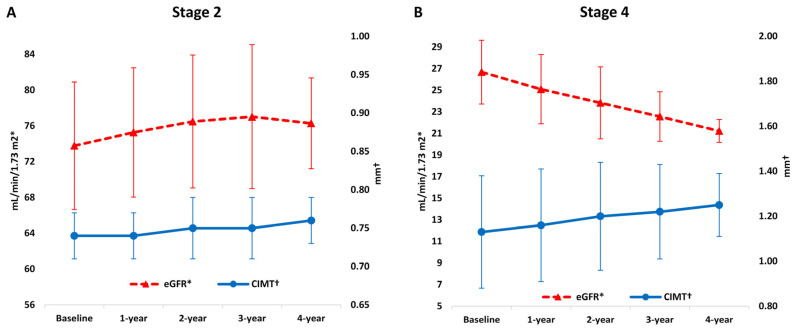
Comparison of eGFR and CIMT values over follow-up: (**A**) Stage 2 of CKD; (**B**) Stage 4 of CKD. Legend: CIMT–carotid intima-media thickness; CKD–chronic kidney disease; eGFR–estimated glomerular filtration rate.

During the follow-up, absolute eGFR values showed a gradual increase in stage 2 (73.78 ± 7.12 vs. 75.27 ± 7.23 vs. 76.49 ± 7.43 vs. 77.02 ± 8.05 vs. 76.28 ± 5.07 mL/min/1.73 m^2^) and gradual decrease in stage 4 CKD group (26.68 ± 2.95 vs. 25.10 ± 3.20 vs. 23.84 ± 3.33 vs. 22.57 ± 2.29 vs. 21.23 ± 1.06 mL/min/1.73 m^2^) (Table 2 and Figure 2), which proved to be statistically significant in the linear mixed effects model (coefficient of 2.51, 3.25, 2.71 and 1.50 for 1-year, 2-year, 3-year and 4-year follow-up, respectively, *P* < 0.05; and a coefficient of −6.69, −5.12, −3.18 and −1.77 for 1-year, 2-year, 3-year and 4-year follow-up, respectively, *P* < 0.001) (Table 3).

Baseline CIMT values were significantly higher in stage 4 compared to stage 2 CKD group (1.13 ± 0.25 vs. 0.74 ± 0.03 mm, *P* < 0.001), and stage 4 CKD group had statistically significantly more atherosclerotic plaques at the study onset (13 (26%) vs. 0 (0%), *P* < 0.001) (Table 1). During the follow-up, absolute CIMT values showed a gradual decrease in stage 4 CKD (1.13 ± 0.25 vs. 1.16 ± 0.25 vs. 1.20 ± 0.24 vs. 1.22 ± 0.21 vs. 1.25 ± 0.14 mm) (Table 2 and Figure 2) which proved to be statistically significant in the linear mixed effects model (coefficient of 0.20, 0.14, 0.07 and 0.03 for 1-year, 2-year, 3-year and 4-year follow-up, respectively, *P* < 0.001) (Table 3). There was no significant temporal change in CIMT values during follow-up in the stage 2 CKD group (Table 3). 

### 3.3. Bivariate Correlation Analysis of eGFR and CIMT Parameters during Follow-Up

**Figure 3:**
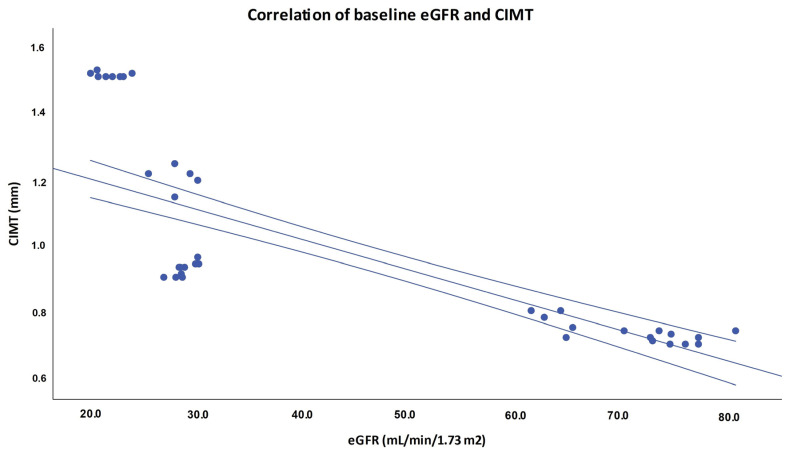
Pearson’s correlation of baseline eGFR and CIMT. Legend: CIMT–carotid intima-media thickness; eGFR–estimated glomerular filtration rate.

There was statistically significant negative correlation of eGFR and CIMT at the beginning of the study and during each time point of the follow-up in both stage 2 and stage 4 CKD group (Table 4 and Figure 3).

### 3.4. Bivariate Correlation Analysis of Selected Baseline Parameters

There was statistically significant negative correlation of baseline eGFR with baseline CIMT (*r* = −0.77, *P* < 0.001), BMI (*r* = −0.63, *P* < 0.001), CRP (*r* = −0.37, *P* < 0.001), PTH (*r* = −0.94, *P* < 0.001) and phosphorus (*r* = −0.67, *P* < 0.001). On the contrary, baseline eGFR exhibited statistically significant positive correlation with cholesterol, calcium and vitamin D levels (*r* = 0.44, *P* < 0.001, *r* = 0.57, *P* < 0.001 and *r* = 0.95, *P* < 0.001, respectively, Table 5).

There was statistically significant positive correlation of baseline CIMT with BMI (*r* = 0.39, *P* < 0.001), cholesterol (*r* = 0.32, *P* = 0.002), CRP (*r* = 0.25, *P* = 0.020), PTH (*r* = 0.72, *P* < 0.001) and phosphorus (*r* = 0.46, *P* < 0.001). On the other hand, baseline CIMT exhibited statistically significant negative correlation with calcium and vitamin D levels (*r* = −0.45, *P* < 0.001 and *r* = −0.73, *P* < 0.001, respectively, Table 5). Other correlation analyses are presented in Table 5.

### 3.5. Multivariable Linear Regression Analysis of Selected Independent Variables in the Prediction of CIMT

The multivariable linear regression model showed that there is a statistically significant independent prediction of CIMT by baseline eGFR (B = −0.85, *P* < 0.001) in the total cohort, while there was no significant prediction of CIMT with other variables (Table 6).

### 3.6. Adverse Events during Follow-Up

**Figure 4:**
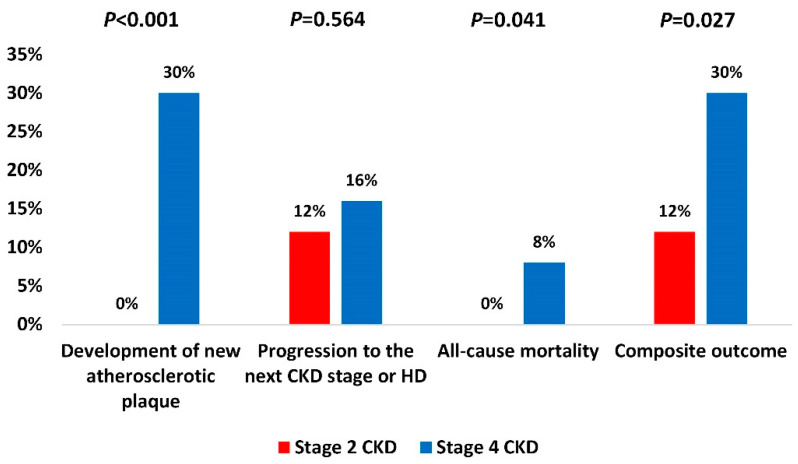
Adverse events during the study period. Chi-square test. Legend: CKD—chronic kidney disease; HD—hemodialysis.

In comparison to stage 2 CKD, stage 4 CKD group had significantly more mortality events (4 (8%) vs. 0 (0%), *P* = 0.041) and more newly developed atherosclerotic plaques (15 (30%) vs. 0 (0%), *P <* 0.001), while there was no statistically significant difference in the rates of progression to the next CKD stage or hemodialysis. Specifically, one patient died in the third year and four patients died in the fourth year of the study with the death cause being acute myocardial infarction and stroke. Finally, stage 4 CKD group had significantly higher occurrence of the composite outcome (15 (30%) vs. 6 (12%), *P* = 0.027) compared to stage 2 CKD (Figure 4).

## 4. Discussion

The current longitudinal study demonstrates a significant association of eGFR and CIMT in non-diabetic patients with stage 2 and stage 4 CKD during the 4-year follow-up. Previous studies have investigated CIMT in relation to renal function in diabetic CKD population [23,24], dialysis patients [15], renal transplant recipients [16] and pediatric population [16,17,18,19,20]. In addition, several studies have enrolled diabetic patients across different spectrums of CKD [5,14,21,22]. However, to the best of our knowledge, there are no longitudinal studies investigating the association of eGFR and CIMT in adult non-diabetic patients with stage 2 and stage 4 CKD over a long-term follow-up. Furthermore, there is limited evidence about CIMT in patients with early CKD stages, who were encompassed in this study.

This study has several major findings. First, non-diabetic patients with stage 4 CKD exhibited significant progressive deterioration of the residual renal function, while the stage 2 CKD group showed dominantly stable function over the 4-year follow-up. Second, non-diabetic stage 4 CKD patients displayed significant gradual expansion of the atherosclerotic burden during the 4-year follow-up as measured by CIMT, whereas stage 2 CKD patients showed minimal non-significant time-dependent CIMT alterations. Furthermore, there was a statistically significant negative correlation of eGFR and CIMT, independently of the study period. Finally, stage 4 CKD patients are significantly more prone to all-cause mortality and the development of new atherosclerotic plaques.

Previous studies have shown differing findings about the association of CIMT and eGFR. Specifically, Chhajed et al. found no statistically significant correlation between CIMT and eGFR in CKD patients [14], while Arroyo et al. found lower CIMT in patients with more advanced CKD [5]. Interestingly, Brito et al. have found strong interaction of CIMT and cystatin C-estimated GFR in African Americans with arterial hypertension, while creatinine-based GFR did not exhibit significant association [21]. The aforementioned studies have partially encompassed diabetic patients (41.4%, 14.4% and 12.6%, respectively) and different CKD stages which complicates direct results comparison [14,21]. In addition, it is also possible that ethnic differences have a role in these discrepancies [5,14,21]. Nevertheless, the current study demonstrated a statistically significant negative correlation of eGFR and CIMT during the 4-year follow-up. Furthermore, this association was independent of the covariates such as age, BMI, systolic blood pressure, LDL-cholesterol and CRP. Consistent findings have been reported by Desbien et al. which demonstrated strong association of CIMT with decreased kidney function [22].

Several other studies have investigated GFR and CIMT in a solely diabetic CKD population. CIMT has shown a strong and independent prediction of cardiovascular mortality in this population, while exhibiting significant negative correlation with eGFR [23,24]. However, diabetes mellitus per se represents a cardiovascular risk factor which triggers the atherosclerosis process and is associated with increased CIMT [27,28]. Therefore, the association of GFR and CIMT cannot be fully elucidated in patients with coexisting CKD and diabetes mellitus [23,24]. More important, a recent meta-analysis discouraged the use of temporal CIMT changes for the measurement of vascular risk in diabetic patients due to lack of association [8]. All aforementioned supports the rationale for the exclusion of diabetic patients in the current longitudinal study. 

The importance of CIMT measurement across different spectrums of CKD has not been fully established. Buscemi et al. have demonstrated a significant negative correlation of GFR and CIMT in patients with near normal or mildly decreased renal function [29], but the impact of further CKD progression has shown differing findings [5,22]. The current study has shown a significant negative correlation of CIMT and eGFR in both stage 2 and stage 4 CKD, but a significantly higher incidence of atherosclerotic plaques in stage 4 CKD during the 4-year period. While the observed stable renal function in stage 2 group could prevent further CIMT progression and plaque development, the study design does not allow causal inferences. Future studies are encouraged to additionally investigate the atherosclerosis process in these patients. Nevertheless, these findings emphasize the need for kidney-sparing measures which could possibly prevent cardiovascular complications, which are the leading cause of mortality in CKD patients [30].

Additionally, stage 4 CKD patients had a significant incidence rate of atherosclerotic plaques during the study period, which are predictive of future cardiovascular events [11]. This finding is important, given the fact that previous studies have questioned the utility of sequential CIMT alterations for the prediction of cardiovascular events [8,11]. Looking at the baseline characteristics, stage 4 CKD patients represented an older population with a higher value of cholesterol and serum phosphate levels which are also predictors of atherosclerosis, as evidenced in the previous studies [31] The importance of a balanced diet, regular physical activity, sleep regulation and smoking cessation should therefore be prioritized [30].

This study has several limitations. A healthy control group could serve as the direct longitudinal comparison with CKD patients, especially with stage 2 CKD, and its absence represents a limitation. Furthermore, some potentially confounding clinical and laboratory parameters were not assessed during the follow-up which prevents longitudinal adjustment analyses, and possible confounding effects could not be eliminated. Moreover, we did not address the different causes of chronic renal failure and the results of our study are not applicable to patients with CKD stages other than 2 and 4. Despite the longitudinal study design, the real causality cannot be established, but rather the association of GFR and CIMT. Finally, this study does not provide insights into the long-term association of GFR and CIMT (>4 years) which should be assessed in different study designs.

## 5. Conclusions

In conclusion, this study demonstrates a significant association of GFR and CIMT in non-diabetic stage 2 and stage 4 CKD patients during the 4-year follow-up. Patients with stage 4 CKD have progressive renal deterioration, higher all-cause mortality and develop new atherosclerotic plaques, in comparison to stage 2 CKD group. Future studies are necessary to determine causality between renal function and atherosclerotic process in these patient groups.

## Figures and Tables

**Table 1:** Comparison of baseline characteristics.

Variables	CKD Stage		Total (*n* = 100)	*P* Value
	Stage 2 (*n* = 50)	Stage 4 (*n* = 50)		
Age (years)	48 (36, 54)	60 (53, 63)	53 (43, 62)	<0.001 ^1^
Male sex	30 (60%)	34 (68%)	64 (64%)	0.405 ^2^
BMI (kg/m^2^)	22.56 ± 2.67	27.46 ± 3.36	25.01 ± 3.90	<0.001 ^3^
Systolic blood pressure (mmHg)	127.80 ± 9.80	149.30 ± 18.24	138.55 ± 18.14	<0.001 ^3^
Diastolic blood pressure (mmHg)	79.92 ± 7.64	82.20 ± 9.75	81.06 ± 8.79	0.196 ^3^
Active smoking	20 (40%)	31 (62%)	51 (51%)	0.085 ^2^
BUN (mmol/L)	5.04 ± 1.35	12.61 ± 1.73	8.83 ± 4.11	<0.001 ^3^
Creatinine (µmol/L)	87.20 ± 12.30	171.48 ± 17.14	129.34 ± 44.88	<0.001 ^3^
eGFR (mL/min/1.73 m^2^)	73.78 ± 7.12	26.86 ± 2.95	50.32 ± 24.19	<0.001 ^3^
CRP (mmol/L)	2.73 ± 1.30	4.04 ± 1.61	3.39 ± 1.60	<0.001 ^3^
Cholesterol (mmol/L)	4.85 ± 1.28	5.85 ± 1.08	5.35 ± 1.28	<0.001 ^3^
HDL cholesterol (mmol/L)	0.89 ± 0.11	1.10 ± 0.16	1.00 ± 0.17	<0.001 ^3^
LDL cholesterol (mmol/L)	2.55 ± 1.03	3.04 ± 0.68	2.80 ± 0.90	0.006 ^3^
Triglycerides (mmol/L)	1.77 ± 0.54	2.09 ± 0.79	1.93 ± 0.69	0.023 ^3^
Serum phosphorus (mmol/L)	1.23 ± 0.07	1.52 ± 0.21	1.37 ± 0.22	<0.001 ^3^
Serum total calcium, corrected (mg/dL)	10.47 ± 0.54	9.85 ± 0.28	10.16 ± 0.53	<0.001 ^3^
Serum PTH (pg/mL)	45.29 ± 5.58	157.65 ± 23.93	101.47 ± 59.05	<0.001 ^3^
25-hydroxyvitamin D (nmol/L)	32.61 ± 2.01	15.81 ± 2.43	24.21 ± 8.73	<0.001 ^3^
Albumin (g/L)	42.96 ± 1.55	39.10 ± 1.15	41.03 ± 2.37	<0.001 ^3^
Carotid intima media thickness (mm)	0.74 ± 0.03	1.13 ± 0.25	0.96 ± 0.27	<0.001 ^3^
Carotid atherosclerotic plaque (>1.5 mm)	0 (0%)	13 (26%)	13 (13%)	<0.001 ^4^

Data are expressed as mean ± SD, number (percent) or median (interquartile range). ^1^ Mann-Whitney U test; ^2^ Chi-square test; ^3^ Student’s T test; ^4^ Fisher’s test. Abbreviations: BMI–body mass index; BUN–blood urea nitrogen; CKD–chronic kidney disease; CRP–C-reactive peptide; eGFR–estimated glomerular filtration rate; HDL–high density lipoprotein; LDL–low density lipoprotein; PTH–parathyroid hormone.

**Table 2:** Absolute eGFR and CIMT values during follow-up.

Variables	Follow-Up ^1^				
	Baseline	1-Year	2-Year	3-Year	4-Year
	**Stage 2**				
eGFR (mL/min/1.73 m^2^)	73.78 ± 7.12	75.27 ± 7.23	76.49 ± 7.43	77.02 ± 8.05	76.28 ± 5.07
CIMT (mm)	0.74 ± 0.03	0.74 ± 0.03	0.75 ± 0.04	0.75 ± 0.04	0.76 ± 0.03
	**Stage 4**				
eGFR (mL/min/1.73 m^2^)	26.68 ± 2.95	25.10 ± 3.20	23.84 ±3.33	22.57 ± 2.29	21.23 ± 1.06
CIMT (mm)	1.13 ± 0.25	1.16 ± 0.25	1.20 ± 0.24	1.22 ± 0.21	1.25 ± 0.14

^1^ Data are derived from descriptive statistics. Data are expressed as mean ± standard deviation. Legend: CIMT—carotid intima-media thickness; eGFR—estimated glomerular filtration rate.

**Table 3:** Change in eGFR and CIMT over follow-up using linear mixed effects model.

Parameters		Parameter Estimates ^1^ (95% CI)	t-Value	*P*	Bayesian Information Criteria
	**Stage 2**				
eGFR	1-year	2.51 (1.10, 3.92)	3.56	0.001	1034.25
	2-year	3.25 (2.47, 4.01)	8.46	<0.001	
	3-year	2.71 (2.17, 3.25)	10.09	<0.001	
	4-year	1.50 (1.24, 1.75)	11.81	<0.001	
CIMT	1-year	0.03 (−0.02, 0.04)	5.02	0.340	−1094.66
	2-year	0.01 (−0.01, 0.02)	4.60	0.061	
	3-year	0.01 (−0.01, 0.01)	2.60	0.073	
	4-year	0.01 (−0.01, 0.02)	1.43	0.160	
	**Stage 4**				
eGFR	1-year	−6.69 (−7.13, −6.25)	−31.79	<0.001	686.67
	2-year	−5.12 (−5.37, −4.87)	−41.02	<0.001	
	3-year	−3.18 (−3.47, −2.89)	−21.96	<0.001	
	4-year	−1.77 (−1.94, −1.60)	−20.94	<0.001	
CIMT	1-year	0.20 (0.17, 0.23)	12.28	<0.001	−516.70
	2-year	0.14 (0.11, 0.17)	9.99	<0.001	
	3-year	0.07 (0.06, 0.09)	9.09	<0.001	
	4-year	0.03 (0.02, 0.03)	8.95	<0.001	

^1^ Baseline values are a reference group. Legend: CI–confidence interval; CIMT–carotid intima-media thickness; eGFR–estimated glomerular filtration rate.

**Table 4:** Pearson’s correlation between selected variables.

r ^1^ (*P* ^2^)				
**Stage 2**				
**Parameters**	**CIMT 1-year**	**CIMT 2-year**	**CIMT 3-year**	**CIMT 4-year**
eGFR 1-year	−0.73 (<0.001)			
eGFR 2-year		−0.74 (<0.001)		
eGFR 3-year			−0.78 (<0.001)	
eGFR 4-year				−0.36 (0.045)
**Stage 4**				
**Parameters**	**CIMT 1-year**	**CIMT 2-year**	**CIMT 3-year**	**CIMT 4-year**
eGFR 1-year	−0.90 (<0.001)			
eGFR 2-year		−0.89 (<0.001)		
eGFR 3-year			−0.71 (<0.001)	
eGFR 4-year				−0.44 (0.006)

^1^ r-value indicates Pearson’s correlation coefficient; ^2^ Pearson’s correlation test. Legend: CIMT—carotid intima-media thickness; eGFR—estimated glomerular filtration rate.

**Table 5:** Pearson’s correlation between selected variables.

r ^1^ (*P* ^2^)									
Parameters	Baseline eGFR	Baseline CIMT	BMI	Cholesterol	CRP	Calcium	25-hydroxyvitamin D	PTH	Phosphorus
Baseline eGFR		−0.77 (<0.001)	−0.63 (<0.001)	0.44 (<0.001)	−0.37 (<0.001)	0.57 (<0.001)	0.95 (<0.001)	−0.94 (<0.001)	−0.67 (<0.001)
Baseline CIMT			0.39 (<0.001)	0.32 (0.002)	0.25 (0.020)	−0.45 (<0.001)	−0.73 (<0.001)	0.72 (<0.001)	0.46 (<0.001)
BMI				0.23 (0.023)	0.27 (0.006)	−0.37 (<0.001)	−0.62 (<0.001)	0.57 (<0.001)	0.50 (<0.001)
Cholesterol					0.11 (0.265)	−0.20 (0.043)	−0.40 (<0.001)	0.33 (0.001)	0.19 (0.059)
CRP						−0.31 (0.002)	−0.40 (<0.001)	0.35 (<0.001)	0.33 (0.001)
Calcium							0.57 (<0.001)	−0.58 (<0.001)	−0.33 (0.001)
25-hydroxyvitamin D								−0.94 (<0.001)	−0.67 (<0.001)
PTH									0.68 (0.001)

^1^ r-value indicates Pearson’s correlation coefficient; ^2^ Pearson’s correlation test. Legend: BMI–body mass index; CIMT–carotid intima-media thickness; CRP–C-reactive protein; eGFR–estimated glomerular filtration rate; PTH—parathyroid hormone.

**Table 6:** Multivariable linear regression analysis of selected parameters as independent predictors of CIMT.

Parameters	B ^1^ (*t* ^2^)	*P*	Overall
Baseline eGFR	−0.85 (−8.27)	<0.001	R^2^ adjusted = 0.590 F ratio = 21.87 *P* < 0.001
Age	0.11 (1.20)	0.235	
BMI	−0.17 (−1.75)	0.085	
Systolic blood pressure	0.03 (0.38)	0.704	
LDL-cholesterol	−0.07 (−0.96)	0.341	
CRP	−0.04 (−0.59)	0.555	

^1^ B-value indicates regression coefficient of the independent variable; ^2^ t-value indicates t-statistic value. Legend: BMI–body mass index; CIMT–carotid intima-media thickness; CRP–C-reactive protein; eGFR–estimated glomerular filtration rate; LDL–low density lipoprotein.

## Data Availability

We disclose any restrictions on the availability of data, materials and associated protocols. Data can be retrieved from the corresponding author at request.
